# Size Matters: d‑Band Holes Drive Plasmonic Chemistry in Gold

**DOI:** 10.1021/acs.nanolett.5c03849

**Published:** 2025-09-22

**Authors:** Quynh Nguyen, Andrea Baldi

**Affiliations:** † 1190 Vrije Universiteit Amsterdam, Department for Physics and Astronomy, De Boelelaan 1100, 1081HZ Amsterdam, The Netherlands

**Keywords:** plasmonics, hot charge carriers, photocatalysis, gold nanoparticle, size dependence

## Abstract

Light absorption in plasmonic gold nanoparticles (AuNPs) generates short-lived nonthermalized electrons and holes that can drive redox reactions, making them attractive for photocatalytic applications. However, characterizing these processes and disentangling competing effects due to photothermal heating or plasmonic field enhancement remain challenging. Here, we exploit the light-driven synthesis of silver shells around AuNPs as a model reaction to study the role of nonthermalized carriers in plasmonic gold photochemistry. By coupling in situ extinction spectroscopy with Mie theory calculations, we extract internal quantum efficiencies (IQEs) for AuNPs with different diameters. The size dependence of the IQE scales with the probability of light absorption within 2–3 nm of the Au nanoparticle surface, highlighting the critical role of short-lived d-band holes, which recombine on ultrafast time scales, in driving our chemistry. More broadly, our approach provides a simple yet powerful framework to characterize activation mechanisms and quantify carrier dynamics in plasmonic photocatalysis.

## Graphical abstract

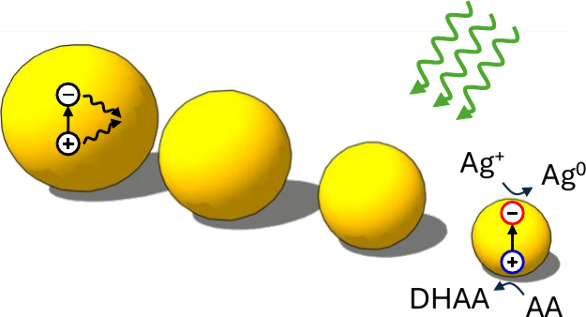

Plasmonic nanomaterials, such as gold nanoparticles (AuNPs), strongly scatter and absorb light thanks to localized surface plasmon resonances (LSPRs), which arise from the collective oscillation of free electrons at the metal surface. Upon excitation, plasmons decay either radiatively (scattering) or nonradiatively (absorption) by generating high-energy electrons and holes.^1–4^ These charge carriers can drive a wide range of photochemical reactions at the surface of the nanoparticles, from water splitting^5–8^ to CO_2_ reduction^9–12^ and nanoparticle synthesis.^13–18^ However, harvesting and utilizing nonequilibrium electrons and holes can be challenging due to their fast thermalization and recombination and, therefore, short lifetimes.^1,19–22^ Although various strategies have been developed to increase their collection efficiency, for example, via engineering of material interfaces^23–29^ or particle geometry,^19,30–34^ a considerable gap in our understanding of the lifetimes and dynamics of plasmonic charge carriers and their contribution to photocatalysis remains.

Several theoretical approaches have been developed to predict the spatial distribution, dynamics, recombination, and transfer of nonthermalized charge carriers in plasmonic nanoparticles.^19,20,22,35^ Transferring this knowledge to experimental settings to predict reactivities and quantum efficiencies of plasmonic photochemical reactions is however challenging. This is due to the presence of complex chemical environments, the intrinsic size and shape heterogeneity of the nanoparticles, the possible existence of competitive reaction pathways, and the highly dynamic nature of the catalytic sites at the particle surface.

In our previous work, we used the light-driven growth of a Ag shell around AuNPs to study the fundamental activation mechanisms of plasmonic chemistry. We have shown that photothermal effects cannot fully explain the observed activity^13^ and found evidence for the role of nonthermalized holes in the electronic d-band of gold as a driver of photochemical reactivity.^40^ Due to their exceedingly short lifetime, the mean free path length of these holes is predicted to be limited to less than 5 nm.^1,36,37^ In this scenario, only d-band holes generated within <5 nm of the nanoparticle surface would reach the interface and participate in redox photoreactions before being thermalized. We therefore hypothesize a strong dependence of photochemical efficiency on the Au core diameter and test this prediction by measuring the rate of Ag shell growth on AuNPs of varying sizes.

We use in situ extinction spectroscopy to measure the rate of Ag shell growth on spherical AuNPs with diameters ranging from 10 to 52 nm. These measurements are carried out both in the dark and under plasmon excitation. By combining our experimental results with Mie theory calculations of the optical properties of Au@Ag core@shell nanoparticles, we extract the internal quantum efficiency (IQE) of the reaction, defined as the number of reduced Ag^+^ ions per absorbed photon, for different Au core diameters. Finally, we demonstrate that the size dependence of the IQE scales as the probability of light absorption within 2–3 nm of the AuNP surface, confirming our hypothesis about the major role of d-band holes in driving the chemical reaction.

We synthesize cetyltrimethylammonium chloride (CTAC)-capped AuNPs with diameters ranging from 10 to 52 nm, using a reported seed-mediated synthesis protocol.^38^ The Au diameters are confirmed via scanning electron microscopy (SEM) (Figure 1a–g), and their mean size is determined via averaging over at least 40 particles with ImageJ (see section S1 of the Supporting Information). For each AuNP size, the total NP concentration is calculated from the optical density (OD) of the final suspension, measured by extinction spectroscopy (details in section S2). The OD of the final suspension is between 6 (smallest AuNPs) and 20 (largest AuNPs). All AuNP suspensions are washed twice by centrifugation and redispersion in 0.1 mM CTAC before being stored in the dark at 4 °C.

**Figure 1:**
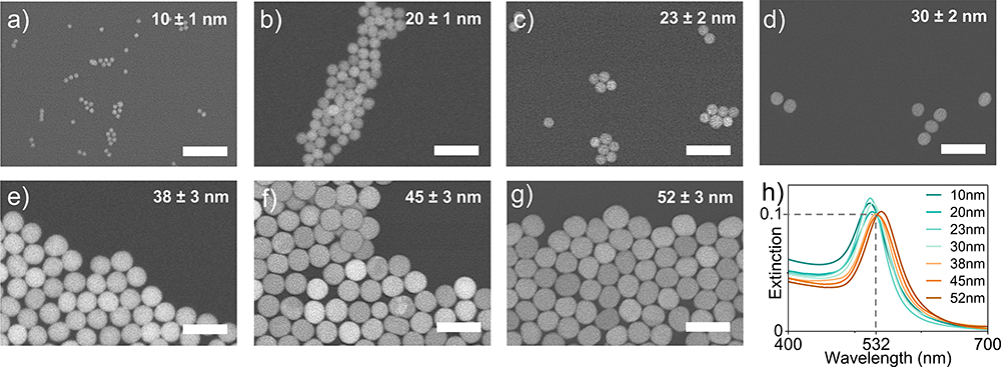
Structural and optical characterization of Au nanoparticles with different diameters. Representative SEM images of AuNPs with average diameters of (a) 10 ± 1, (b) 20 ± 1, (c) 23 ± 2, (d) 30 ± 2, (e) 38 ± 3, (f) 45 ± 3, and (g) 52 ± 3 nm. Scale bars are 100 nm. (h) Extinction spectra of aqueous suspensions of the AuNPs in panels a–g diluted to an optical density of 0.1 at an excitation wavelength of 532 nm (dashed lines).

We characterize the rate of Ag shell growth around Au cores with different diameters both in the dark and under irradiation with a collimated 532 nm continuous wave (CW) laser. The laser power is kept at 160 mW with a beam width of ∼1 mm. All reaction solutions are diluted with 0.1 mM CTAC to obtain an optical density of 0.1 at 532 nm at the beginning of the reaction (Figure 1h) and kept under stirring at a constant temperature of 20 °C. At these irradiation intensities and nanoparticle concentrations, both localized and collective heating effects are negligible.^39^ The CTAC concentration used is high enough to guarantee NP stability while preventing the formation of light-scattering byproducts that were observed in control experiments above the critical micellar concentration of 1.2 mM.

As schematically illustrated in Figure 2a, the Ag shell growth is initiated by sequentially adding bis­(*p*-sulfonatophenyl)­phenylphosphine (BSPP, 375 mM, 51 μL), silver nitrate (AgNO_3_, 150 mM, 51 μL), and l-ascorbic acid (AA, 150 mM, 154 μL) under continuous stirring to 1744 μL of an aqueous suspension of the AuNPs, yielding a total reaction volume of 2 mL with an OD of 0.1 at 532 nm. In this reaction, AgNO_3_ serves as the silver precursor, AA acts as a mild reducing agent, and BSPP coordinates the Ag^+^ ions forming a soluble Ag^+^–BSPP complex that buffers the free Ag^+^ concentration in solution.^13,40^ CTAC serves as a surfactant, maintaining the colloidal stability of the AuNPs throughout the reaction.

**Figure 2:**
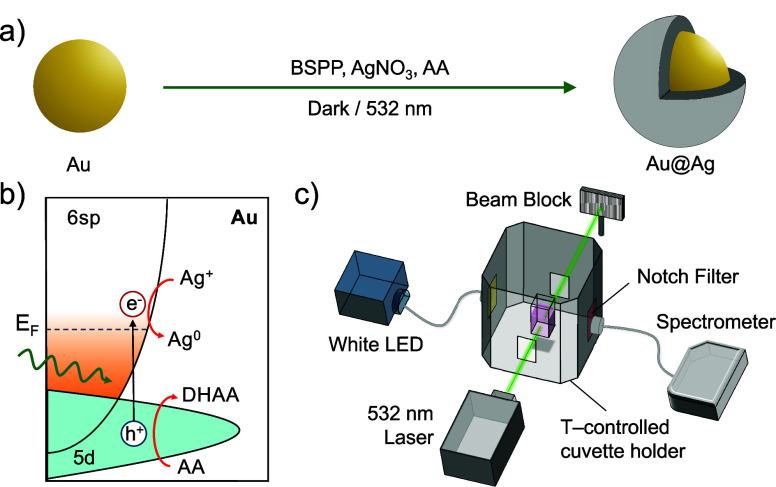
Photochemical Ag shell growth and experimental setup. (a) Schematic of the light-driven synthesis of Au@Ag core@shell nanoparticles in the presence of BSPP, AgNO_3_, and AA. (b) Proposed reaction mechanism in which interband excitation in Au generates d-band holes that oxidize AA to dehydroascorbic acid (DHAA), releasing two electrons that reduce Ag^+^ to Ag^0^ at the NP surface. (c) Experimental setup for in situ extinction measurements. The reaction mixture in a quartz cuvette is either kept in the dark or irradiated with a 532 nm CW laser, while extinction spectra are recorded orthogonally using a white light LED source (Ocean Optics HL-2000-FHGA) and a fiber-coupled spectrometer (Ocean Optics Flame Miniature). A 532 ± 9 nm notch filter protects the spectrometer from scattered laser light, and a 360 nm long-pass filter cuts off any UV component from the LED. The reaction mixture is continuously stirred and maintained at 20 °C using a temperature-controlled cuvette holder (Quantum Northwest Qpod 3).

For each AuNP size, we initially measure the rate of Ag shell growth in the dark for 1 h. The dark rate is then subtracted from the average one measured under laser irradiation to properly account for the intrinsic activity of the AuNPs when determining the IQE of the photochemical reaction. We then perform at least three independent 1 h long irradiation measurements for each AuNP diameter.

The Ag shell growth mechanism is hypothesized to proceed via interband light absorption in Au, leading to a highly energetic hole in the d-band and a nearly thermalized electron close to the Fermi level. Light absorption is then followed by the fast oxidation of AA by the holes reaching the Au surface and the subsequent reduction of Ag^+^ to Ag^0^ (Figure 2b). The reaction is monitored in real time via extinction spectroscopy in a 3 mL quartz cuvette (path length of 10 mm × 10 mm), with spectra recorded every 15 s under continuous stirring at 400 rpm and 20 °C in a temperature-controlled cuvette holder (Figure 2c). The cuvette was covered with a Teflon cap to prevent evaporation.

Control experiments in the dark reveal a small baseline reactivity (Figure 3a), while laser excitation induces a pronounced increase in the extinction in the blue region as well as a blue-shift and intensity increase in the LSPR, consistent with Ag shell formation (Figure 3b). To quantify these changes, we simulate extinction spectra of Au cores with varying Ag shell thicknesses, using Mie theory and the dielectric functions both provided by Johnson and Christy (Figure 3c).^41,42^

**Figure 3:**
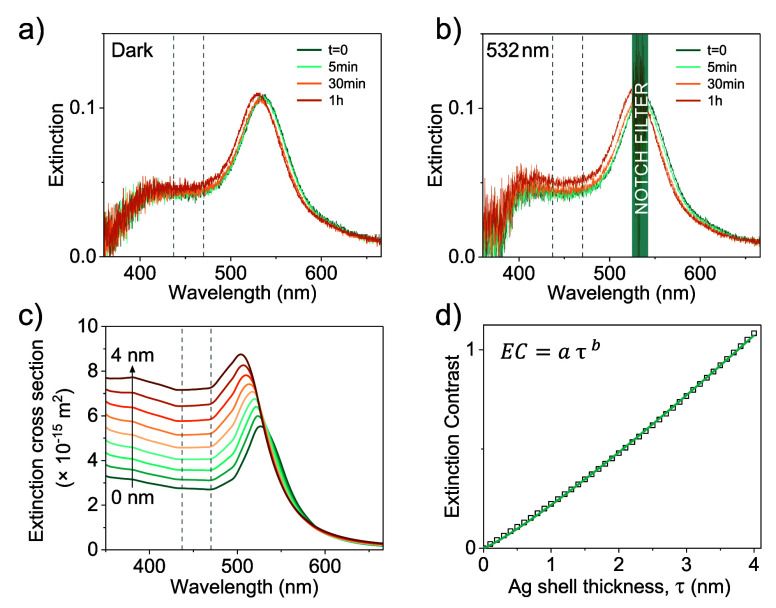
Extinction spectra of reaction solutions containing 45 nm AuNPs, BSPP, AgNO_3_, and AA, measured over 1 h (a) in the dark and (b) under 532 nm laser irradiation (160 mW, 1 mm 1/e^2^ Gaussian beam diameter). (c) Mie theory-calculated extinction cross sections of Au@Ag core@shell particles with a 45 nm core and increasing Ag shell thicknesses. The dashed lines in panels a–c mark the spectral region (430–470 nm) used for calculating the EC. (d) Calculated extinction contrast vs Ag shell thickness for Au@Ag NPs with a 45 nm diameter Au core. The green line is a fit to the data using eq 2.

We define the internal quantum efficiency of the reaction as the number of Ag^+^ ions reduced per photon absorbed. To calculate the IQE, we begin by extracting the growth rate of the Ag shell from our extinction spectra via integration of the overall extinction intensity between 430 and 470 nm. This spectral region is sufficiently blue-shifted from the Au LSPR while being strongly influenced by the presence of a Ag shell. As in our previous work,^13^ we quantify this signal by defining the extinction contrast (EC) that captures the relative change in extinction over this spectral window with respect to the spectrum at time zero:

1 $$\mathrm{EC}\left(t\right)=\frac{\int_{430\;\mathrm{nm}}^{470\;\mathrm{nm}}\mathrm{Ext}\left(t,\lambda \right)\;d\lambda -\int_{430\;\mathrm{nm}}^{470\;\mathrm{nm}}\mathrm{Ext}\left(0,\lambda \right)\;d\lambda }{\int_{430\;\mathrm{nm}}^{470\;\mathrm{nm}}\mathrm{Ext}\left(0,\lambda \right)\;d\lambda }$$

To link the EC signal to the Ag^+^ reduction kinetics, we generate synthetic EC curves from simulated extinction spectra of Au@Ag NPs with increasing Ag shell thicknesses (Figure 3d). For each core diameter, the simulated relationship between EC and Ag shell thickness τ is fitted to the empirical relationship

2 $$\mathrm{EC}=a\tau^{b}$$

where *a* and *b* are fit parameters. During the laser irradiation experiments, for each AuNP diameter, we irradiate the suspension for 1 h and measure the evolution of the EC. By inverting eq 2, we then determine the thickness of the Ag shell, τ, which allows us to deduce the rate of the Ag^+^ reduction reaction. The shell volume in a Au@Ag particle is

3 $$V_{\mathrm{shell}}=\frac{4}{3}\pi {\left(R+\tau \right)}^{3}-\frac{4}{3}\pi R^{3}$$

where *R* is the radius of the Au core. The number of Ag^+^ ions reduced per particle in during a 1 h illumination is therefore

4 $$n_{{Ag}^{+}}=\rho_{\mathrm{Ag}}V_{\mathrm{shell}}$$

where ρ_Ag_ = 58.55 atoms/nm^3^ is the silver atomic density. The Ag^+^ reduction rate in units of Ag^+^ ions per nanoparticle per second, corrected by the reduction rate during 1 h under dark conditions, is therefore

5 $$r_{Ag^{+}\;\mathrm{reduction}}=\frac{n_{{Ag}^{+}}-n_{{Ag}^{+}}\left(\mathrm{dark}\right)}{3600\;(s)}\;({\mathrm{Ag}}^{+}\;\mathrm{ions reduced/NP/s)}$$

To determine the quantum efficiency of the photochemical process, we calculated the number of absorbed photons per particle per second. The photon energy for a wavelength of 532 nm is

6 $$E_{\mathrm{photon}}=\frac{hc}{\lambda }=\frac{6.626\times 10^{-34}\;(\mathrm{J s})\times 2.998\times 10^{8}\;(m/s)}{532\times 10^{-9}\;(m)}=3.73\times 10^{-19}\;(J)$$

The incident photon rate from the laser with a power *P*_laser_ of 160 mW is therefore

7 $$I_{\mathrm{incident}}=\frac{P_{\mathrm{laser}}}{E_{\mathrm{photon}}}=\frac{0.16\;(J/s)}{3.73\times 10^{-19}\;(J/\mathrm{photon})}=4.3\times 10^{17}\;(\mathrm{incident photons/s)}$$

Since all of our AuNP suspensions have an optical density of 0.1 at the laser wavelength, the number of extinguished photons per second through our cuvette is constant and given by

8 $$I_{\mathrm{ext}}=I_{\mathrm{incident}}\left(1-T\right)=I_{\mathrm{incident}}\left({1-10}^{-\mathrm{OD}}\right)=4.3\times 10^{17}\;(\mathrm{incident photons/s)}\times \left({1-10}^{-0.1}\right)=8.8\times 10^{16}\;(\mathrm{extinguished photons/s)}$$

The number of extinguished photons per second includes photons absorbed by the nanoparticles, which can give rise to photochemical reactions as well as scattered photons that do not contribute to it. Assuming a negligible contribution of multiple scattering events, the number of photons absorbed per second can be calculated from the relative contribution of the absorption (σ_abs_) and extinction (σ_ext_) cross sections of our AuNPs prior to Ag shell growth at 532 nm:

9 $$I_{abs}=I_{\mathrm{ext}}\times \frac{\sigma_{\mathrm{abs}}}{\sigma_{\mathrm{ext}}}\;(\mathrm{absorbed photons/s)}$$

The absorption and extinction cross sections are calculated from Mie theory using literature values for Au permittivity.^41,42^ Mie theory calculations of Au@Ag core@shell particles show a slight decrease in the absorption cross section at 532 nm with an increase in shell thickness. However, the extinction contrasts at the end of our reactions suggest that the Ag shells remain thinner than ∼1.5 nm for all of the Au sizes explored. For this reason, we use the absorption cross section of the bare Au cores as a reasonable approximation when evaluating the absorbed power during the entire reaction. The number of absorbed photons per second per nanoparticle is obtained by dividing *I*_abs_ by the number of nanoparticles (*N*_NP_) in the cuvette (see also section S2):

10 $$r_{\mathrm{photon absorption}}=\frac{I_{abs}}{N_{\mathrm{NP}}}\;(\mathrm{absorbed photons/NP/s)}$$

The IQE is finally defined as

11 $$\mathrm{IQE}=\frac{r_{Ag^{+}\;\mathrm{reduction}}\;({\mathrm{Ag}}^{+}\;\mathrm{ions reduced/NP/s)}}{r_{\mathrm{photon absorption}}\;(\mathrm{absorbed photons/NP/s)}}$$

A detailed summary of all kinetic and optical parameters used in the IQE calculations, as well as all the EC versus time trends measured for each AuNP core diameter, is provided in section S3.

By applying this approach to all investigated core diameters, we found a pronounced size dependence of the IQE for the Ag^+^ reduction under green laser excitation (Figure 4a). In particular, the IQE decreases with an increase in Au core diameter, with the smallest AuNPs (10 nm) exhibiting the highest efficiency.

**Figure 4:**
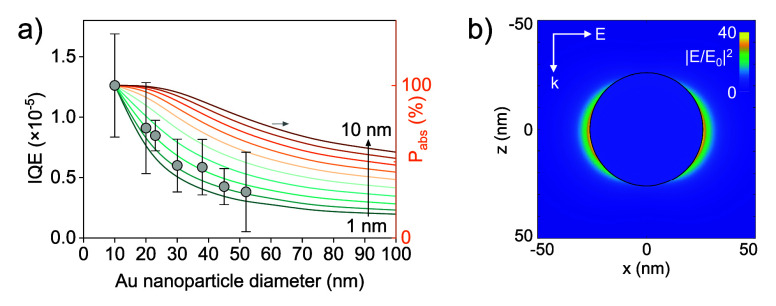
(a) Internal quantum efficiency measured for the Ag^+^ reduction reaction as a function of AuNP diameter (left axis). The error bar represents the standard deviation over at least three independent measurements. Colored lines (right axis) indicate the percentage of optical power absorbed within the indicated distance of the AuNP surface, normalized by the value for a Au particle with a diameter of 10 nm. The absorbed power is calculated from Mie theory. (b) Calculated electric field intensity enhancement |*E*/*E*_0_|^2^ inside and outside of a 52 nm AuNP in the *x–z* plane.

While larger particles absorb more incident light, their lower IQE indicates that their photochemical enhancement is less efficient than for smaller particles under these conditions. In smaller NPs, the higher surface-to-volume ratio increases the probability that photogenerated hot electrons and holes reach the nanoparticle/water interface before undergoing energy relaxation via electron–phonon or electron–electron scattering. In contrast, as the Au core diameter increases, a larger fraction of hot carriers is generated farther from the particle surface, increasing their likelihood of recombining within the particle, before contributing to interfacial redox chemistry. This scenario is consistent with recent studies on size-dependent hot-carrier generation, which demonstrate that only carriers generated within a certain distance of the surface can effectively participate in surface reactions.^31,43^

An even more quantitative analysis of carrier dynamics was attempted by quantifying the spatial distribution of absorbed power within the nanoparticles, as shown in the Mie theory calculations in Figure 4a (colored curves). For each particle diameter, the internal electric field intensity enhancement, |*E*/*E*_0_|^2^, was computed on a three-dimensional grid spanning the nanoparticle volume at an excitation wavelength of 532 nm, following the formalism described by Bohren and Huffman.^44^ The power loss density *P*_loss_(*r*) is calculated as

12 $$P_{\mathrm{loss}}\left(r\right)=0.5\varepsilon_{0}\omega \varepsilon_{2}\left(\lambda \right){\left|E\left(r\right)/E_{0}\right|}^{2}$$

where ε_2_ is the imaginary part of the Au dielectric function and *E*(*r*) is the field amplitude inside the particle (Figure 4b). Note that Figure 4b shows the field intensity enhancement both outside the NP, with pronounced field localization originating from the dipolar LSPR, and inside the NP, where the field intensity is significantly lower and nearly homogeneous. By integrating the power loss density over concentric shells from the particle surface inward, we determined the fraction of total absorbed power within a selected distance (between 1 and 10 nm) of the surface for different AuNP diameters. In Figure 4a, we plot the size dependence of the power absorbed within a selected distance, normalized by the value calculated for a 10 nm AuNP. The IQE of our photochemical reaction displays the same size dependence as the power absorbed (i.e., the number of nonthermalized electrons and holes generated) in a region within 2–3 nm of the particle surface. This range is fully consistent with the predicted mean free paths of nonthermalized d-band holes (<5 nm), confirming our original hypothesis that these charge carriers drive plasmonic chemistry in gold under green light irradiation.

Interestingly, we also observe that for larger, strongly faceted AuNPs the IQE increases again, significantly deviating from the monotonic size trend observed for spherical particles (section S4). Given the known influence of particle faceting on their photochemical properties,^45–49^ it is perhaps unsurprising to observe a strong shape dependence also in our Ag shell growth reaction. The higher IQE for faceted AuNPs may be associated with enhanced field localization and hence d-band hole generation in the vicinity of the particle surface.

In summary, we used optical spectroscopy to determine the internal quantum efficiency of a plasmon-driven photochemical reaction on Au nanoparticles. We find that small spherical nanoparticles outperform larger ones in utilizing absorbed photons to drive chemistry. The size dependence of the internal quantum efficiency provides conclusive evidence for the major role of nonthermalized holes in the gold’s electronic d-band in driving the observed photochemical process. Together, these results provide new design principles for next-generation plasmonic photocatalysts and broaden our understanding of hot-carrier utilization in plasmon-driven chemistry. More broadly, our work demonstrates that quantitative information about internal quantum efficiencies and hot-carrier dynamics can be derived from straightforward steady-state spectroscopy coupled to electron microscopy and simple analytical modeling. In this respect, our study provides an accessible framework for the characterization of photochemical rates in plasmon-mediated reactions.

## Supplementary Material



## Notes

Nanoparticle size distribution (section S1), calculation of nanoparticle concentrations (section S2), IQE calculation parameters (section S3), and size-dependent IQE and the role of particle faceting (section S4) (PDF)

## Abbreviations

AuNPs: gold nanoparticles
LSPRs: localized surface plasmon resonances
CW: continuous wave
SEM: scanning electron microscopy
OD: optical density
CTAC: cetyltrimethylammonium chloride
BSPP: bis­(*p*-sulfonatophenyl)­phenylphosphine
AA: l-ascorbic acid
AgNO_3_: silver nitrate
IQE: internal quantum efficiency
NP: nanoparticle
