# Supplementary material for: Size Matters: d‑Band Holes Drive Plasmonic Chemistry in Gold
Source: Nano Lett. 2025 Sep 22;25(40):14704–9. doi: 10.1021/acs.nanolett.5c03849 (PMC12512193; doi:10.1021/acs.nanolett.5c03849)
Supplement: Supplementary file 1 [file nl5c03849_si_001.pdf]

Supporting Information

Size Matters: d-band Holes Drive Plasmonic Chemistry in Gold

Quynh Nguyen[1], Andrea Baldi[1]*

Vrije Universiteit Amsterdam, Department for Physics and Astronomy, De Boelelaan 1100, 1081HZ Amsterdam, the Netherlands

*corresponding author: a.baldi@vu.nl

Table of Contents

1. Nanoparticle size distribution 3

2. Calculation of nanoparticle concentration 4

3. IQE calculation parameters 5

4. Size-dependent IQE and the role of particle faceting 8

References 9

1. Nanoparticle size distribution

The size distributions of all NP batches used in this study were determined scanning electron microscopy image analysis. For each batch, the diameters of at least 40 individual nanoparticles were measured using ImageJ, and the results are presented as histograms in Figure S1.

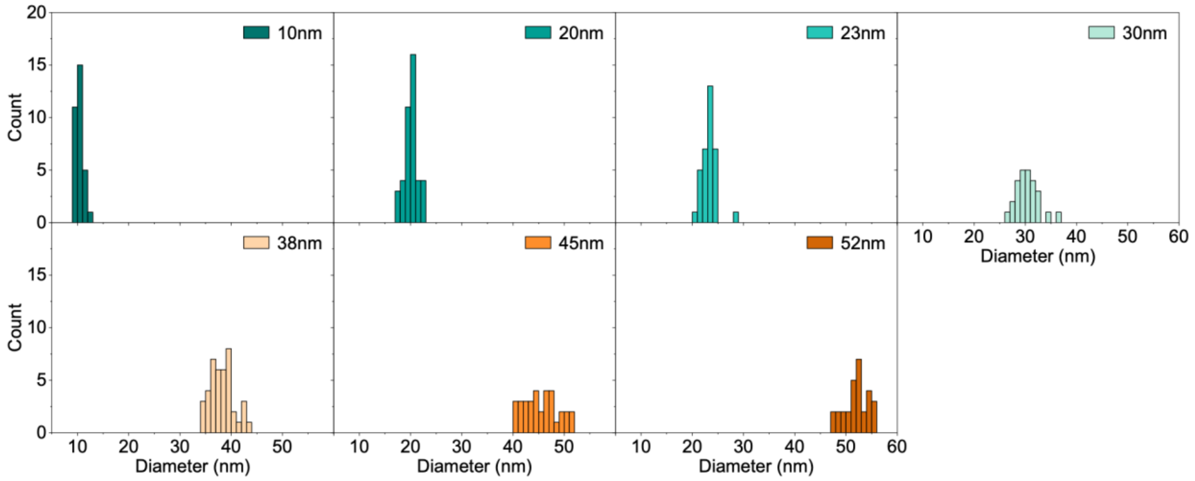

Figure S1. Size distribution histograms for Au nanoparticles of different core diameters, determined by image analysis of SEM images.

2. Calculation of nanoparticle concentration

The nanoparticle concentration (c_{NP} in NP/ml) is calculated using the Beer-Lambert law:

$$c_{NP} = \frac{\ln(10) \times OD}{\sigma_{ext} \times l} \times 10^{-6} \quad (1)$$

where OD is the optical density (or extinction) at the LSPR position of the Au suspension, σ_{ext} is the extinction cross section at the same wavelength (m^2/NP), and l is the optical path length in the cuvette (m).

The extinction cross sections for AuNPs of different diameters are calculated from Mie theory,[1] using literature values for the Au permittivity.[2] The calculations are performed using Matlab script freely available at https://github.com/andrea-baldi/Mie_Scattering_and_Absorption_Sphere

The total number of particles $N_{NP,}$ in the reaction solution with a total Volume V_{tot} is therefore:

$$N_{NP} = c_{NP} \times V_{tot} \quad (2)$$

The absorption cross section at 532 nm of the most absorbing Au NPs in the present study (i.e. the largest Au NPs with a diameter of 52 nm) is ~7,500 nm^2. This absorption cross section is nearly identical to the one of the Au nanorods used in our previous ACS Energy Letters at 730 nm (~7,700 nm^2),[3] for which we calculated and measured completely negligible localized and collective photothermal effects. While the power of the 532 nm laser used in this study (160 mW) is ~3x larger than the one of the 730 nm laser used in our previous study (55 mW), the particle concentration here ($2.66 \cdot 10^9$ NPs/ml) is ~3x smaller ($8.2 \cdot 10^9$ NPs/ml), so the expected collective heating effect is comparable and therefore also negligible.

3. IQE calculation parameters

A summary of all parameters used in the calculation of the IQE for each AuNP core diameter is provided in Table S1. For each size, we report the empirical fit parameters a and b from the relation $EC = a\tau^b$, the average Ag^+ reduction rate per nanoparticle, the total number of nanoparticles in the reaction volume, the measured absorbance at 532 nm, the calculated photon absorption and the resulting internal quantum efficiency.

For each AuNP core diameter, one measurement was performed in the dark (listed as Dark in Table S1), while subsequent entries (numbered 1, 2, etc.) correspond to independent experiments carried out under continuous 532 laser illumination at 160 mW. The reported IQE values are dark-corrected averages and standard deviations derived from light driven experiments.

All extracted extinction contrast curves are shown in Figure S2. In all our measurements, we observe an initial induction period of up to ~15 min during which the extinction contrast remains roughly constant or increases slowly, followed by a faster Ag shell growth. This behavior has also been observed in our previous work,[3] and may be related to a slight difference in the rate of Ag^+ reduction on the fresh Au surface at the beginning of the reaction versus the Ag surface exposed after a few minutes of shell growth.

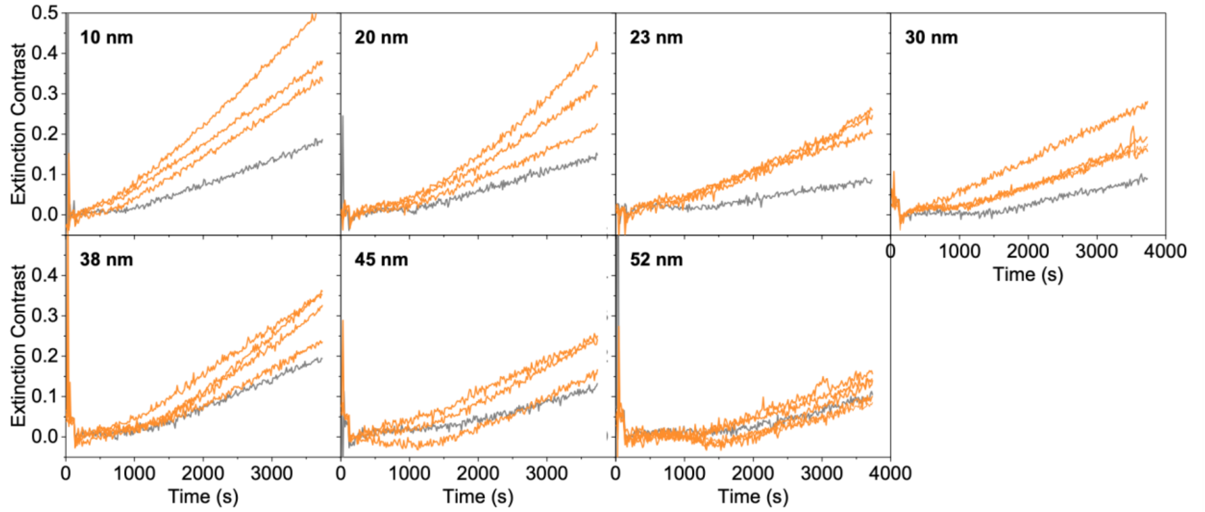

Figure S2. Extinction contrast curves for Au@Ag core shell nanoparticle synthesis on 10-52 nm diameter Au cores. Orange lines represent reactions performed under 532 nm laser illumination, grey lines represent reactions performed under dark conditions.

Control experiments were performed on AuNP suspensions in 0.1 mM CTAC under 532 nm laser irradiation to assess sample photostability (Figure S3). No spectral changes are observed over 1h of illumination, indicating that the laser irradiation does not induce any photothermal

damage, shape deformation, or nanoparticle aggregation.

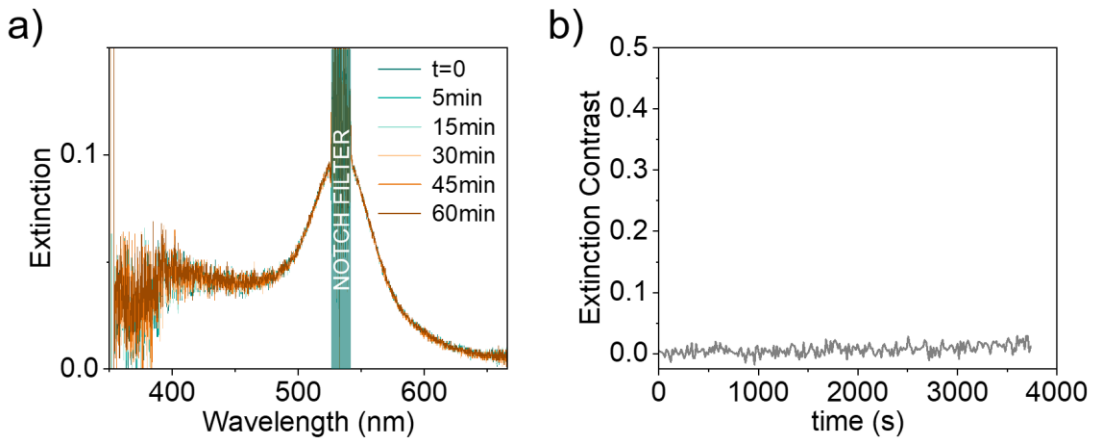

Figure S3. (a) Extinction spectra of a reaction solution containing 38 nm AuNPs suspended in 0.1 mM CTAC, measured under 532 nm laser irradiation over 1h. (b) Corresponding extinction contrast curve. The y-axis range is the same as in Figure S2.

Table S1 Parameters used in the IQE calculations.

AuNP diameter (nm)	Fit parameter a	Fit parameter b	Reaction	Ag reduction rate (Ag/NP/s)	NPs	$\frac{\sigma_{abs}}{\sigma_{ext}}$ at 532 nm	Photons absorbed per particle (photons/NP/s)	IQE
10	1.20148	1.20544	Dark	1.1681	9.74E+11	0.998	9.02E+04	1E-05 ± 4E-06
			1	2.1756				
			2	1.9146				
			3	2.8306				
20	0.49455	1.37322	Dark	9.0137	1.12E+11	0.99	7.80E+05	9.10E-06 ± 3.76E-06
			1	16.1618				
			2	12.4923				
			3	19.6768				
23	0.46144	1.19032	Dark	6.8547	7.59E+10	0.985	1.14E+06	8.48E-06 ± 1.26E-06
			1	14.547				
			2	17.89				
			3	17.2121				
30	0.3481	1.1439	Dark	14.8376	3.15E+10	0.968	2.70E+06	6.01E-06 ± 2.18E-06
			1	26.8481				
			2	27.2963				
			3	28.9588				
			4	41.2172				
38	0.25981	1.41286	Dark	61.3376	1.44E+10	0.938	5.72E+06	5.87E-06 ± 2.29E-06
			1	72.8729				
			2	97.1293				
			3	105.78				
			4	103.8777				
45	0.22477	1.08386	Dark	70.8866	8.44E+09	0.903	9.42E+06	

			1	91.2873				4.26E-06 ± 1.49E-06
			2	118.8425				
			3	122.873				
52	0.18392	1.1151	Dark	84.9195	5.32E+09	0.858	1.42E+07	3.82E-06 ± 3.29E-06
			1	212.01				
			2	87.1996				
			3	154.86				
			4	116.43				
			5	125.38				
69	0.12576	1.03062	Dark	128.52	2.67E+09	0.730	2.41E+07	1.17E-05 ± 3.52E-06
			1	492.1251				
			2	444.54				
			3	293.1607				
86	0.08674	1.04664	Dark	706	1.93E+09	0.601	5.29E+16	2.37E-05 ± 3.94E-06
			1	1284.4				
			2	1272.6				
			3	1506.8				

4. Size-dependent IQE and the role of particle faceting

We investigated the photochemical IQE of AuNPs with diameters larger than 50 nm (69 and 86 nm). The extracted IQEs deviate from the monotonic decrease of smaller spherical particles (Figure S4a). Structural characterization via SEM reveals that these larger particles exhibit strong faceting and polyhedral shapes, in contrast to the spherical morphology of AuNPs in the 10-52 nm range (Figure S4b-c).

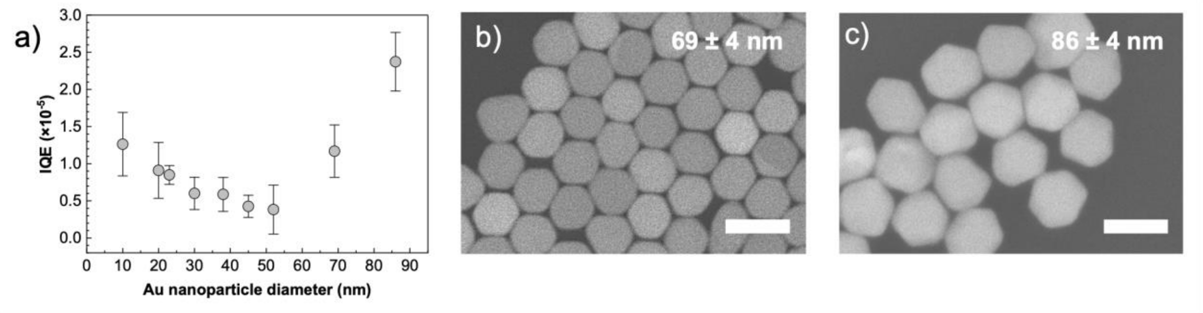

Figure S4. (a) Internal quantum efficiency for Ag^{+} reduction as a function of Au nanoparticle core diameter. (b) SEM images of AuNPs with average diameters of 69 nm and (c) 86 nm. All scalebars are 100 nm.

References

(1) Mie, G. Beiträge Zur Optik Trüber Medien, Speziell Kolloidaler Metallösungen. *Annalen der Physik* **1908**, *330* (3), 377–445. https://doi.org/10.1002/andp.19083300302.

(2) Johnson, P. B.; Christy, R. W. Optical Constants of the Noble Metals. *Phys. Rev. B* **1972**, *6* (12), 4370–4379. https://doi.org/10.1103/PhysRevB.6.4370.

(3) Kamarudheen, R.; Aalbers, G. J. W.; Hamans, R. F.; Kamp, L. P. J.; Baldi, A. Distinguishing Among All Possible Activation Mechanisms of a Plasmon-Driven Chemical Reaction. *ACS Energy Lett.* **2020**, *5* (8), 2605–2613. https://doi.org/10.1021/acsenergylett.0c00989.

(4) Kamarudheen, R.; Castellanos, G. W.; Kamp, L. P. J.; Clercx, H. J. H.; Baldi, A. Quantifying Photothermal and Hot Charge Carrier Effects in Plasmon-Driven Nanoparticle Syntheses. *ACS Nano* **2018**, *12* (8), 8447–8455. https://doi.org/10.1021/acsnano.8b03929.
